# Ubiquitin System

**DOI:** 10.3390/ijms19041080

**Published:** 2018-04-04

**Authors:** Nobuhiro Nakamura

**Affiliations:** Department of Life Science and Technology, Tokyo Institute of Technology, 4259-B13 Nagatsuta-cho, Midori-ku, Yokohama 226-8501, Japan; nnakamur@bio.titech.ac.jp; Tel.: +81-45-924-5726

Ever since the discovery of ubiquitin in 1975 [1], extensive research studies have shown that the ubiquitin system is essential for maintaining homeostasis of the body by controlling a wide range of cellular functions. Dysfunction of the ubiquitin system leads to various human diseases, particularly neurodegenerative disorders [2]. The ubiquitin system is, therefore, gaining attention as a promising drug target. However, the mechanisms behind the physiological and pathophysiological actions of the ubiquitin system are not fully understood owing to its diverse and complicated nature. First, there exists a large number of substrate proteins and ubiquitin ligases (E3s) (more than 600 in humans) responsible for substrate recognition. Second, the fate of substrate proteins is largely determined by multiple types of ubiquitin conjugation, such as monoubiquitination, multiubiquitination, and polyubiquitination with at least eight different linkages [3]. Third, ubiquitination can be modulated by ~100 deubiquitinating enzymes (DUBs), which remove or trim ubiquitin chains on substrate proteins. To further understand the cellular roles and the underlying mechanisms of ubiquitination, it is necessary not only to identify substrates of E3s and DUBs but also to determine the type(s) and effect(s) of ubiquitin conjugation on each substrate protein. The aim of this special issue “Ubiquitin System” is to increase knowledge about the biochemical, structural, and pathophysiological aspects of the ubiquitin system. Of the 25 submissions received, 18 papers were selected and published after rigorous peer review.

Several articles highlight the importance of ubiquitination in the regulation of intracellular signaling pathways and their cellular functions. Although transforming growth factor-beta (TGF-β) signaling has tumor-suppressive effects in normal and premalignant cells, it promotes tumorigenesis and metastasis in the late stage of tumor progression. Many studies have investigated the mechanism underlying the switch of TGF-β signaling from a tumor suppressor to an oncogene. Iyengar [4] reviewed the recent advances in elucidating the ubiquitin system’s role in regulating TGF-β signaling. Several E3s and DUBs play crucial roles in both the canonical (SMAD-dependent) and non-canonical (SMAD-independent) TGF-β pathways, and their activities are controlled through ubiquitination, phosphorylation, neddylation, and sumoylation. The DUB ubiquitin-specific protease 15 (USP15) is one such regulator of TGF-β signaling, and its structural and functional properties are summarized in the review article by Chou et al. [5]. UPS15 has two opposing functions: on the one hand, it has an oncogenic effect by activating TGF-β signaling and stabilizing the E3 ubiquitin ligase MDM2, but on the other hand, it also acts as a tumor suppressor by negatively regulating the Wnt pathway through constitutive photomorphogenesis 9 (COP9) signalosome (CSN)-associated deubiquitination. Given these conflicting functions, further study is needed to determine the precise roles of USP15 and its underlying mechanisms in related physiological and pathophysiological conditions. Mitogen-activated protein kinase (MAPK) signaling is also regulated by ubiquitination. MEKK1, a MAPK kinase, activates the extracellular signal-regulated kinase (ERK) and c-Jun N-terminal kinase (JNK) pathways by phosphorylating mitogen-activated protein kinase kinase (MKK)1 and MKK4. However, MEKK1 also acts as an E3 and negatively regulates these phosphorylation events by undergoing self-ubiquitination, although its functional relevance is uncertain [6]. Zhang et al. [7] reported that a recombinant staphylokinase variant fusion protein (SAK-HV) decreases MEKK1 self-ubiquitination and promotes macrophage proliferation through activation of the ERK and JNK pathways. They identified the E1 ubiquitin-activating enzyme UBA1 as a binding partner for SAK-HV and proposed an attractive working model in which SAK-HV blocks the interaction of UBA1 with an E2 ubiquitin-conjugating enzyme(s) that mediates MEKK1 self-ubiquitination. Telesio et al. [8] identified ring finger protein 7 (RNF7) as a novel regulator of nuclear factor-kappa B (NF-κB) signaling. RNF7 interacts with CARMA2*sh*, the short isoform of caspase recruitment domain-containing protein 14 (CARMA2/CARD14), and inhibits CARMA2*sh-*mediated NF-κB activation. Interestingly, RNF7 reduces the ubiquitination of both mucosa-associated lymphoid tissue lymphoma translocation protein 1 (MALT1) and NF-κB essential modulator (NEMO) without affecting their protein levels. Considering the fact that RNF7 is an E3, the reduced ubiquitination is most likely to be an indirect effect of RNF7, but the underlying mechanism remains unclear. Sánchez-Sánchez and Arévalo [9] summarized the current understanding of the regulation of neurotrophin receptor signaling by ubiquitination and discussed its clinical relevance. Ubiquitination also contributes to the desensitization and downregulation of G protein-coupled receptors (GPCRs) [10]. This topic has been extensively studied and reviewed in yeast and mammals. The review article by Pergolizzi et al. [11] for the first time summarizes the recent advances in GPCR (especially cAMP receptors) signaling in the social amoeba *Dictyostelium discoideum* and involvement of the ubiquitin system in its regulation. As the evolutionarily-conserved components of GPCR signaling and the ubiquitin system exist in *Dictyostelium*, this amoeba has the potential to serve as a useful model organism to investigate the ubiquitin-mediated regulation of GPCR signaling.

Ubiquitination is important for maintaining cellular homeostasis, as well as coping with cellular stress and damage. Medina et al. [12] reported that mitochondrial DNA (mtDNA) depletion increases the uptake of amino acids in the human osteosarcoma cell line. They also found in an earlier study that amino acid transporters (solute carrier (SLC) transporters) are deubiquitinated in the mtDNA-depleted cells [13]. It is possible that deubiquitination of the amino acid transporters might control the cellular transport activity of amino acids in response to the cellular energy status. Yokoe and Asahi [14] identified phospholamban (PLN) as a novel ubiquitination substrate of the von Hippel-Lindau protein (pVHL) E3 complex. PLN plays an important role in cardiac contractility by inhibiting sarcoplasmic reticulum Ca^2+^ ATPase (SERCA2a). pVHL is upregulated, whereas PLN is ubiquitinated and downregulated, in the heart of mice with genetically dilated cardiomyopathy, as well as in response to HEK293 cells undergoing oxidative stress. pVHL may contribute to heart failure in response to oxidative stress. The review article by Dubrez [15] provides a comprehensive overview of the ubiquitin-mediated regulation of the E2F1 transcription factor during cell cycle progression and in DNA damage response. It is noteworthy that E2F1 undergoes K11, K48, K63, and K11/K48 branch-linked ubiquitination in a cell-cycle phase-dependent manner. In this regard, Beclin 1, a key regulator of autophagy, is modified by distinct types of ubiquitin chains to control autophagy pathways, including autophagosome formation, which is reviewed by Boutouja et al. [16]. Ubiquitin is an evolutionarily-conserved protein, the functionality of which was explained in the paper of Allan and Phillips through their analysis using thermodynamic scaling [17]. Thus, polyubiquitin chains can be formed through one of the seven lysine residues or the first methionine residue of ubiquitin in a variety of species. The function of ubiquitination is specified by its conjugation pattern as well as its conjugation site on a target protein. However, researchers face difficulty in investigating the biological properties and functions of each ubiquitination because a sufficient amount of ubiquitinated proteins cannot be produced enzymatically with E1, E2, and E3. The review article by Morimoto et al. [18] focuses on the chemical ubiquitination methods developed to overcome the above problem. In some cases, ubiquitination occurs at non-lysine sites (i.e., Ser, Thr, and Cys) in substrate proteins, although the biological significance of non-canonical ubiquitination is not clear [19]. Sánchez-Lanzas and Castaño [20] reported that lysine-deficient mutants of the survival motor neuron gene products SMN and SMN∆7 undergo proteasomal degradation, but interestingly their degradation does not require N-terminal or non-canonical ubiquitination.

The ubiquitin system is involved in the pathogenesis of autoimmune and inflammatory diseases, such as arthritis, psoriasis, and allergy [21]. The case report by Rossi et al. [22] described a correlation between autoimmune uveitis and high serum levels of ubiquitin and proteasome. Tsuchida et al. [23] summarized the roles of the ubiquitin system in periodontal disease characterized by inflammation of the gum tissue. Ubiquitination regulates proinflammatory signaling, including NF-κB signaling, in response to bacterial infections of the gum tissue. Wilck et al. [24] investigated the effect of low-dose treatment with the proteasome inhibitor bortezomib on advanced atherosclerosis in low-density lipoprotein receptor (LDLR)-deficient mice. They showed that low-dose bortezomib does not affect aortic atherosclerotic lesions or serum levels of inflammatory markers and increases plaque instability. Given the fact that low-dose bortezomib treatment attenuates early atherosclerosis in LDLR-deficient mice [25], further study is needed to analyze the dose- and stage-dependent effects, as well as the underlying mechanisms of proteasome inhibition on atherosclerosis. The proteasome is a widely recognized therapeutic target in inflammatory and autoimmune diseases. Proteasome inhibition is also important for fatty liver graft preservation by cold storage. Panisello-Roselló et al. [26] evaluated the relevance of the ubiquitin system in cytoprotection during cold storage using Institut Georges Lopez (IGL)-1 and histidine-tryptophan-ketoglutarate (HTK) solutions. The IGL-1 solution provided a better cytoprotective effect on fatty liver grafts than did the HTK solution; this is likely to be through low ubiquitin–proteasome activity, increased endothelial nitric oxide synthase (e-NOS) expression, and AMP kinase (AMPK) signaling. Mikamo et al. [27] investigated the role of S-phase kinase-associated protein 2 (Skp2), an E3 that regulates the cell cycle, in pulmonary fibrosis using Skp2-deficient mice. Targeted disruption of Skp2 attenuated bleomycin-induced pulmonary fibrosis and downregulation of p27, a substrate of Skp2. The same inhibitory effect was obtained by treatment of wild-type mice with the Skp2 inhibitor SZL-P1-41, suggesting that p27 degradation has a role in the progression of pulmonary fibrosis and Skp2 is a potential therapeutic target for this disease.

The functions and regulatory mechanisms of ubiquitination are becoming increasingly complicated owing to the identification of novel substrates, regulators, and ubiquitin chain patterns (e.g., branched ubiquitination). All the papers in this special issue provide new insights into the biochemical, pathological, and therapeutic aspects of the ubiquitin system. I would like to express my gratitude to all the authors for their contribution to this special issue and to all the reviewers for their helpful and insightful comments on the papers.
